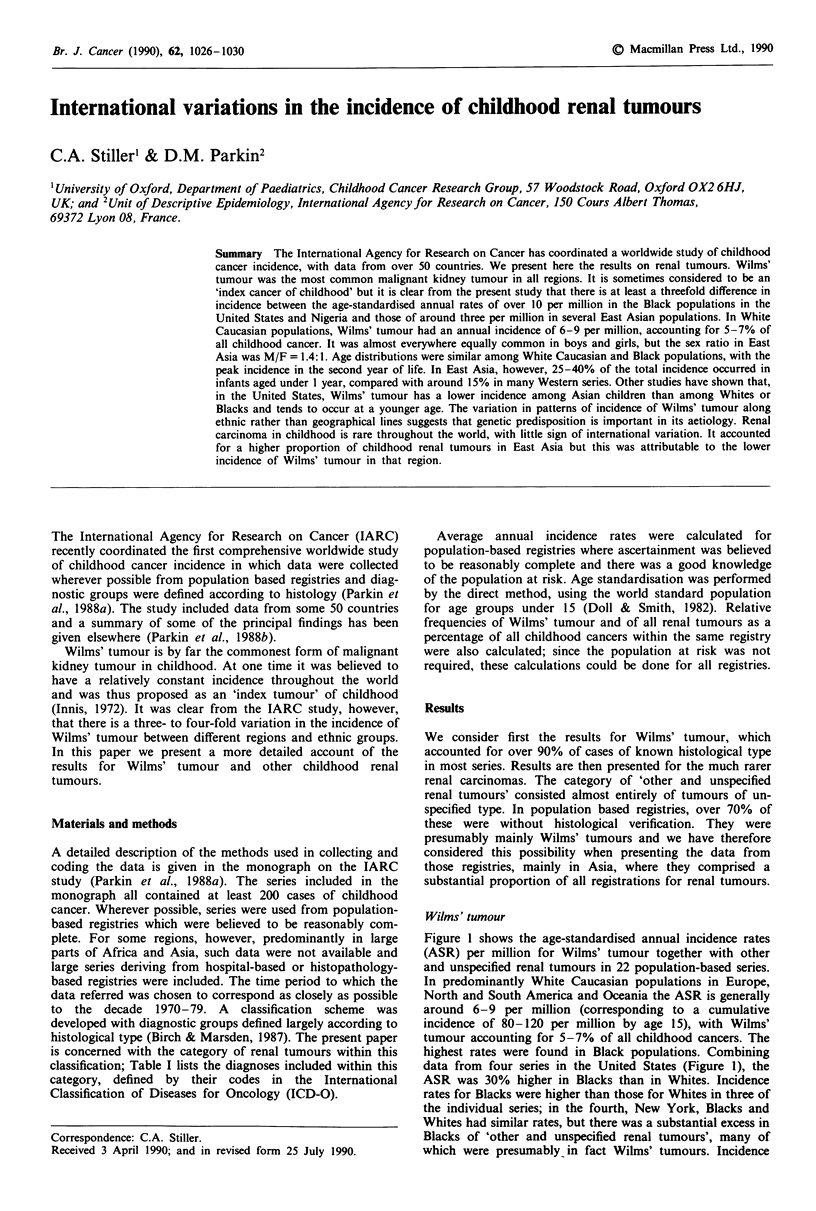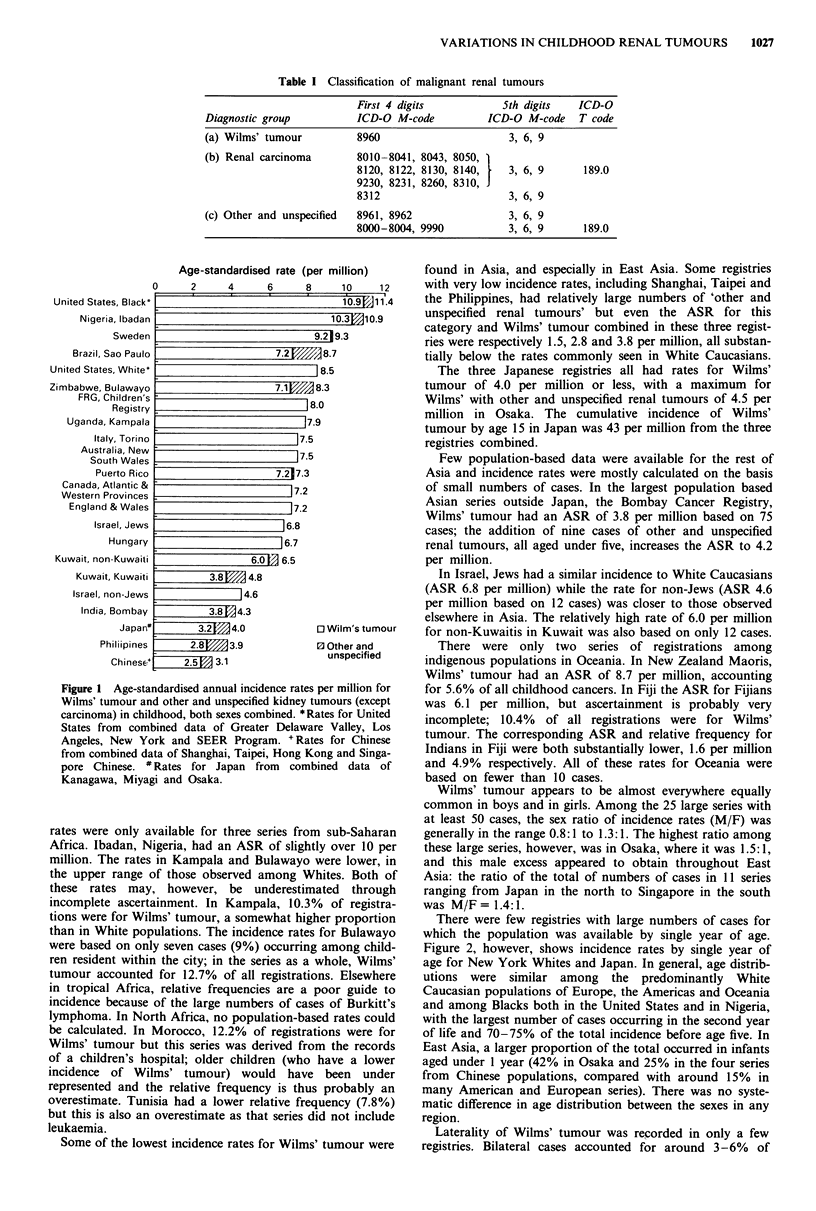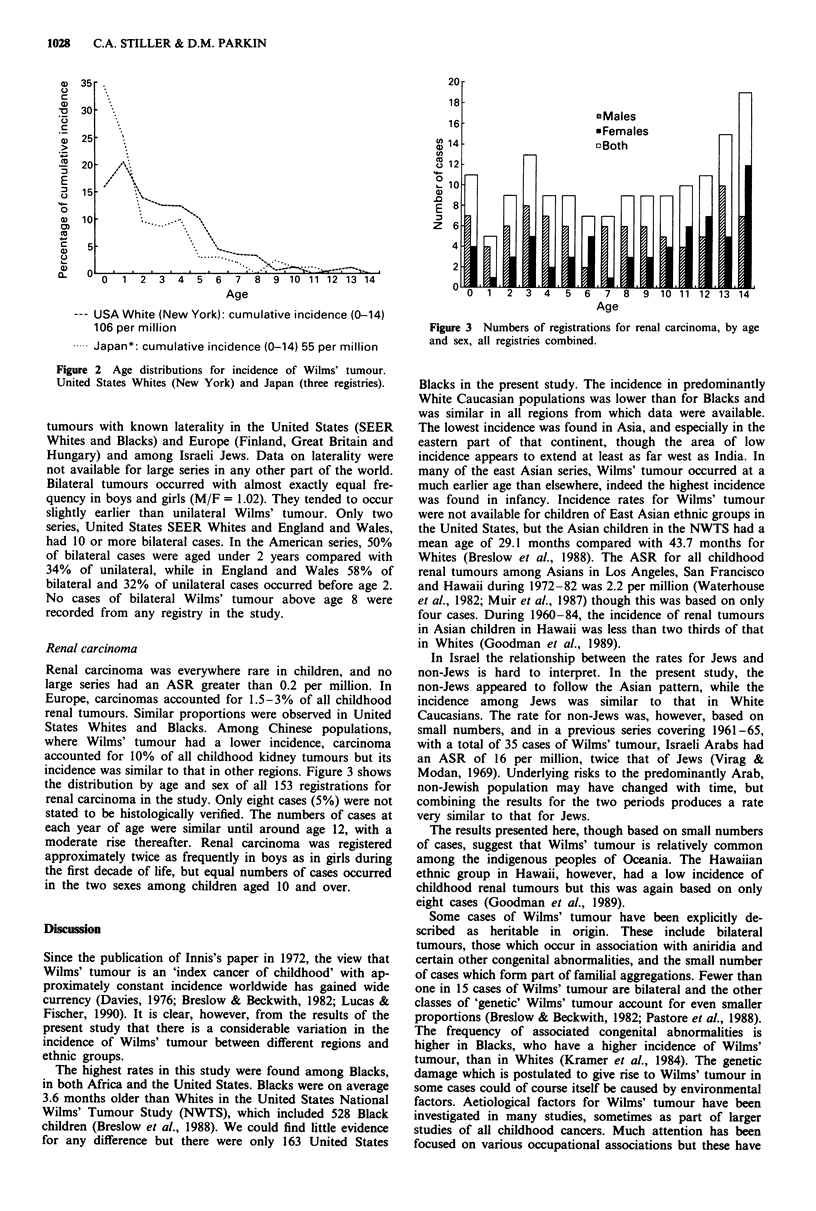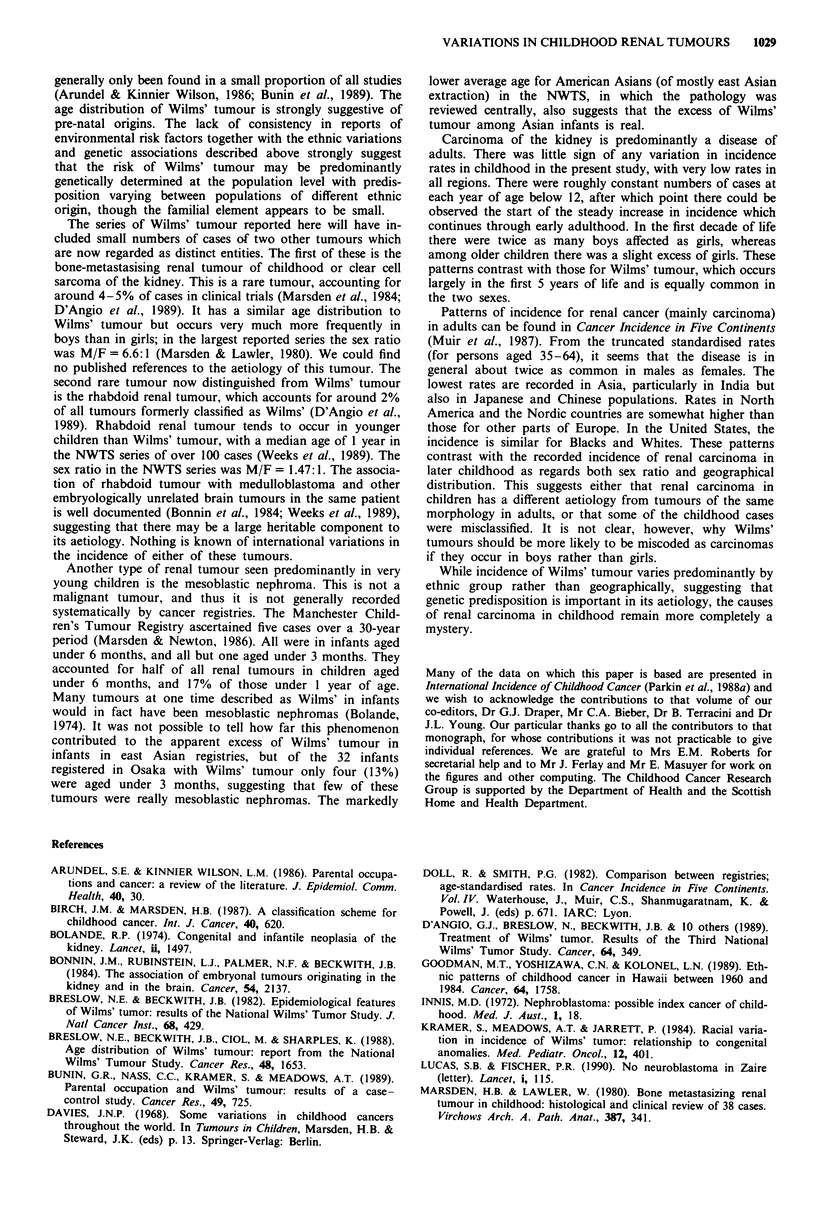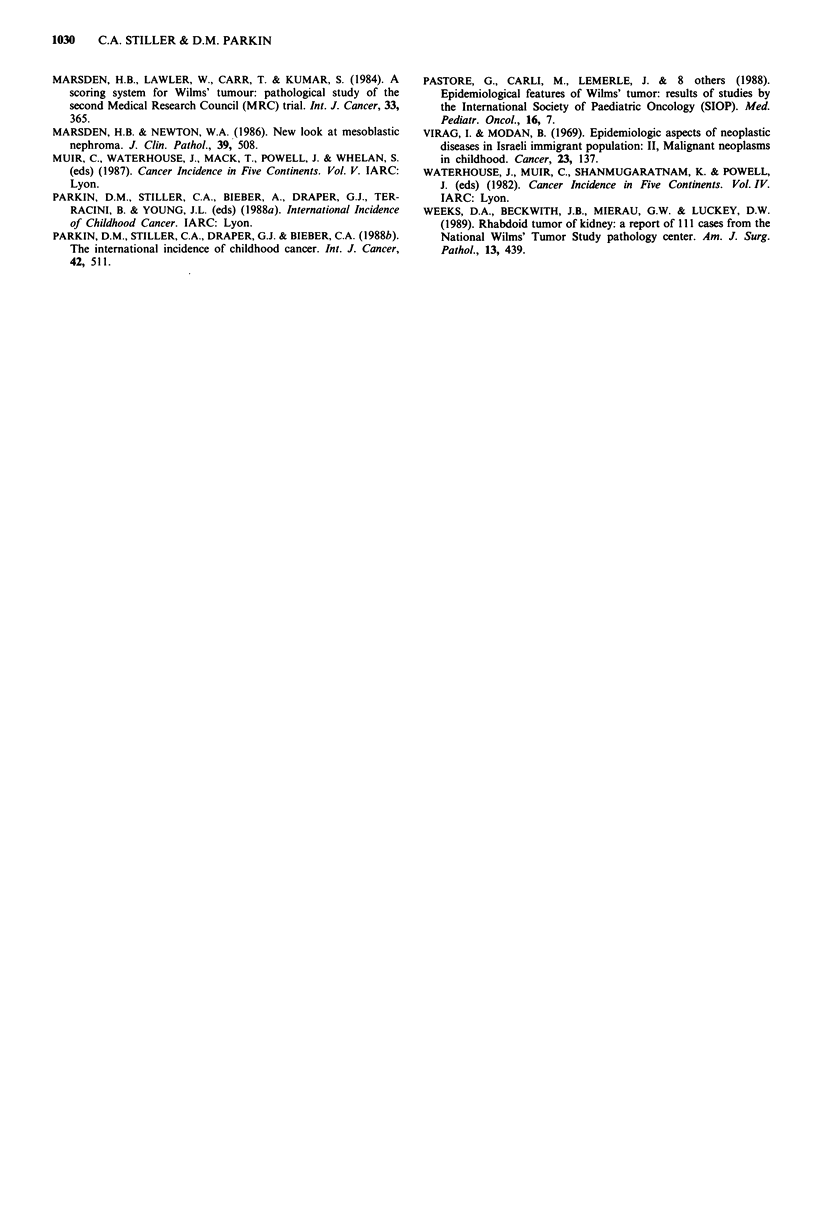# International variations in the incidence of childhood renal tumours.

**DOI:** 10.1038/bjc.1990.432

**Published:** 1990-12

**Authors:** C. A. Stiller, D. M. Parkin

**Affiliations:** University of Oxford, Department of Paediatrics, UK.

## Abstract

The International Agency for Research on Cancer has coordinated a worldwide study of childhood cancer incidence, with data from over 50 countries. We present here the results on renal tumours. Wilms' tumour was the most common malignant kidney tumour in all regions. It is sometimes considered to be an 'index cancer of childhood' but it is clear from the present study that there is at least a threefold difference in incidence between the age-standardised annual rates of over 10 per million in the Black populations in the United States and Nigeria and those of around three per million in several East Asian populations. In White Caucasian populations, Wilms' tumour had an annual incidence of 6-9 per million, accounting for 5-7% of all childhood cancer. It was almost everywhere equally common in boys and girls, but the sex ratio in East Asia was M/F = 1.4:1. Age distributions were similar among White Caucasian and Black populations, with the peak incidence in the second year of life. In East Asia, however, 25-40% of the total incidence occurred in infants aged under 1 year, compared with around 15% in many Western series. Other studies have shown that, in the United States, Wilms' tumour has a lower incidence among Asian children than among Whites or Blacks and tends to occur at a younger age. The variation in patterns of incidence of Wilms' tumour along ethnic rather than geographical lines suggests that genetic predisposition is important in its aetiology. Renal carcinoma in childhood is rare throughout the world, with little sign of international variation. It accounted for a higher proportion of childhood renal tumours in East Asia but this was attributable to the lower incidence of Wilms' tumour in that region.


Br. J. Cancer (1990), 62, 1026-1030 © Macmillan Press Ltd., 1990

### International variations in the incidence of childhood renal tumours

C.A. Stiller[1] & D.M. Parkin[2]

[1]*University of Oxford, Department of Paediatrics, Childhood Cancer Research Group, 57 Woodstock Road, Oxford OX2 6HJ, UK; and* [2]*Unit of Descriptive Epidemiology, International Agency for Research on Cancer, 150 Cours Albert Thomas, 69372 Lyon 08, France.*

**Summary** The International Agency for Research on Cancer has coordinated a worldwide study of childhood cancer incidence, with data from over 50 countries. We present here the results on renal tumours. Wilms' tumour was the most common malignant kidney tumour in all regions. It is sometimes considered to be an 'index cancer of childhood' but it is clear from the present study that there is at least a threefold difference in incidence between the age-standardised annual rates of over 10 per million in the Black populations in the United States and Nigeria and those of around three per million in several East Asian populations. In White Caucasian populations, Wilms' tumour had an annual incidence of 6-9 per million, accounting for 5-7% of all childhood cancer. It was almost everywhere equally common in boys and girls, but the sex ratio in East Asia was M/F = 1.4:1. Age distributions were similar among White Caucasian and Black populations, with the peak incidence in the second year of life. In East Asia, however, 25-40% of the total incidence occurred in infants aged under 1 year, compared with around 15% in many Western series. Other studies have shown that, in the United States, Wilms' tumour has a lower incidence among Asian children than among Whites or Blacks and tends to occur at a younger age. The variation in patterns of incidence of Wilms' tumour along ethnic rather than geographical lines suggests that genetic predisposition is important in its aetiology. Renal carcinoma in childhood is rare throughout the world, with little sign of international variation. It accounted for a higher proportion of childhood renal tumours in East Asia but this was attributable to the lower incidence of Wilms' tumour in that region.

The International Agency for Research on Cancer (IARC) recently coordinated the first comprehensive worldwide study of childhood cancer incidence in which data were collected wherever possible from population based registries and diagnostic groups were defined according to histology (Parkin *et al.*, 1988*a*). The study included data from some 50 countries and a summary of some of the principal findings has been given elsewhere (Parkin *et al.*, 1988*b*).

Wilms' tumour is by far the commonest form of malignant kidney tumour in childhood. At one time it was believed to have a relatively constant incidence throughout the world and was thus proposed as an 'index tumour' of childhood (Innis, 1972). It was clear from the IARC study, however, that there is a three- to four-fold variation in the incidence of Wilms' tumour between different regions and ethnic groups. In this paper we present a more detailed account of the results for Wilms' tumour and other childhood renal tumours.

### Materials and methods

A detailed description of the methods used in collecting and coding the data is given in the monograph on the IARC study (Parkin *et al.*, 1988*a*). The series included in the monograph all contained at least 200 cases of childhood cancer. Wherever possible, series were used from population-based registries which were believed to be reasonably complete. For some regions, however, predominantly in large parts of Africa and Asia, such data were not available and large series deriving from hospital-based or histopathology-based registries were included. The time period to which the data referred was chosen to correspond as closely as possible to the decade 1970-79. A classification scheme was developed with diagnostic groups defined largely according to histological type (Birch & Marsden, 1987). The present paper is concerned with the category of renal tumours within this classification; Table I lists the diagnoses included within this category, defined by their codes in the International Classification of Diseases for Oncology (ICD-O).

Average annual incidence rates were calculated for population-based registries where ascertainment was believed to be reasonably complete and there was a good knowledge of the population at risk. Age standardisation was performed by the direct method, using the world standard population for age groups under 15 (Doll & Smith, 1982). Relative frequencies of Wilms' tumour and of all renal tumours as a percentage of all childhood cancers within the same registry were also calculated; since the population at risk was not required, these calculations could be done for all registries.

### Results

We consider first the results for Wilms' tumour, which accounted for over 90% of cases of known histological type in most series. Results are then presented for the much rarer renal carcinomas. The category of 'other and unspecified renal tumours' consisted almost entirely of tumours of unspecified type. In population based registries, over 70% of these were without histological verification. They were presumably mainly Wilms' tumours and we have therefore considered this possibility when presenting the data from those registries, mainly in Asia, where they comprised a substantial proportion of all registrations for renal tumours.

### *Wilms' tumour*

Figure 1 shows the age-standardised annual incidence rates (ASR) per million for Wilms' tumour together with other and unspecified renal tumours in 22 population-based series. In predominantly White Caucasian populations in Europe, North and South America and Oceania the ASR is generally around 6-9 per million (corresponding to a cumulative incidence of 80-120 per million by age 15), with Wilms' tumour accounting for 5-7% of all childhood cancers. The highest rates were found in Black populations. Combining data from four series in the United States (Figure 1), the ASR was 30% higher in Blacks than in Whites. Incidence rates for Blacks were higher than those for Whites in three of the individual series; in the fourth, New York, Blacks and Whites had similar rates, but there was a substantial excess in Blacks of 'other and unspecified renal tumours', many of which were presumably in fact Wilms' tumours. Incidence

Correspondence: C.A. Stiller.
Received 3 April 1990; and in revised form 25 July 1990.

**Table I** Classification of malignant renal tumours

| Diagnostic group | First 4 digits ICD-O M-code | 5th digits ICD-O M-code | ICD-O T code |
|---|---|---|---|
| (a) Wilms' tumour | 8960 | 3, 6, 9 | |
| (b) Renal carcinoma | 8010–8041, 8043, 8050, 8120, 8122, 8130, 8140, 9230, 8231, 8260, 8310, | 3, 6, 9 | 189.0 |
| | 8312 | 3, 6, 9 | |
| (c) Other and unspecified | 8961, 8962 | 3, 6, 9 | |
| | 8000–8004, 9990 | 3, 6, 9 | 189.0 |

**Figure 1** Age-standardised annual incidence rates per million for Wilms' tumour and other and unspecified kidney tumours (except carcinoma) in childhood, both sexes combined. *Rates for United States from combined data of Greater Delaware Valley, Los Angeles, New York and SEER Program. +Rates for Chinese from combined data of Shanghai, Taipei, Hong Kong and Singapore Chinese. #Rates for Japan from combined data of Kanagawa, Miyagi and Osaka.

rates were only available for three series from sub-Saharan Africa. Ibadan, Nigeria, had an ASR of slightly over 10 per million. The rates in Kampala and Bulawayo were lower, in the upper range of those observed among Whites. Both of these rates may, however, be underestimated through incomplete ascertainment. In Kampala, 10.3% of registrations were for Wilms' tumour, a somewhat higher proportion than in White populations. The incidence rates for Bulawayo were based on only seven cases (9%) occurring among children resident within the city; in the series as a whole, Wilms' tumour accounted for 12.7% of all registrations. Elsewhere in tropical Africa, relative frequencies are a poor guide to incidence because of the large numbers of cases of Burkitt's lymphoma. In North Africa, no population-based rates could be calculated. In Morocco, 12.2% of registrations were for Wilms' tumour but this series was derived from the records of a children's hospital; older children (who have a lower incidence of Wilms' tumour) would have been under represented and the relative frequency is thus probably an overestimate. Tunisia had a lower relative frequency (7.8%) but this is also an overestimate as that series did not include leukaemia.

Some of the lowest incidence rates for Wilms' tumour were found in Asia, and especially in East Asia. Some registries with very low incidence rates, including Shanghai, Taipei and the Philippines, had relatively large numbers of 'other and unspecified renal tumours' but even the ASR for this category and Wilms' tumour combined in these three registries were respectively 1.5, 2.8 and 3.8 per million, all substantially below the rates commonly seen in White Caucasians.

The three Japanese registries all had rates for Wilms' tumour of 4.0 per million or less, with a maximum for Wilms' with other and unspecified renal tumours of 4.5 per million in Osaka. The cumulative incidence of Wilms' tumour by age 15 in Japan was 43 per million from the three registries combined.

Few population-based data were available for the rest of Asia and incidence rates were mostly calculated on the basis of small numbers of cases. In the largest population based Asian series outside Japan, the Bombay Cancer Registry, Wilms' tumour had an ASR of 3.8 per million based on 75 cases; the addition of nine cases of other and unspecified renal tumours, all aged under five, increases the ASR to 4.2 per million.

In Israel, Jews had a similar incidence to White Caucasians (ASR 6.8 per million) while the rate for non-Jews (ASR 4.6 per million based on 12 cases) was closer to those observed elsewhere in Asia. The relatively high rate of 6.0 per million for non-Kuwaitis in Kuwait was also based on only 12 cases.

There were only two series of registrations among indigenous populations in Oceania. In New Zealand Maoris, Wilms' tumour had an ASR of 8.7 per million, accounting for 5.6% of all childhood cancers. In Fiji the ASR for Fijians was 6.1 per million, but ascertainment is probably very incomplete; 10.4% of all registrations were for Wilms' tumour. The corresponding ASR and relative frequency for Indians in Fiji were both substantially lower, 1.6 per million and 4.9% respectively. All of these rates for Oceania were based on fewer than 10 cases.

Wilms' tumour appears to be almost everywhere equally common in boys and in girls. Among the 25 large series with at least 50 cases, the sex ratio of incidence rates (M/F) was generally in the range 0.8:1 to 1.3:1. The highest ratio among these large series, however, was in Osaka, where it was 1.5:1, and this male excess appeared to obtain throughout East Asia: the ratio of the total of numbers of cases in 11 series ranging from Japan in the north to Singapore in the south was M/F = 1.4:1.

There were few registries with large numbers of cases for which the population was available by single year of age. Figure 2, however, shows incidence rates by single year of age for New York Whites and Japan. In general, age distributions were similar among the predominantly White Caucasian populations of Europe, the Americas and Oceania and among Blacks both in the United States and in Nigeria, with the largest number of cases occurring in the second year of life and 70–75% of the total incidence before age five. In East Asia, a larger proportion of the total occurred in infants aged under 1 year (42% in Osaka and 25% in the four series from Chinese populations, compared with around 15% in many American and European series). There was no systematic difference in age distribution between the sexes in any region.

Laterality of Wilms' tumour was recorded in only a few registries. Bilateral cases accounted for around 3–6% of

**Figure 2** Age distributions for incidence of Wilms' tumour. United States Whites (New York) and Japan (three registries).

**Figure 3** Numbers of registrations for renal carcinoma, by age and sex, all registries combined.

tumours with known laterality in the United States (SEER Whites and Blacks) and Europe (Finland, Great Britain and Hungary) and among Israeli Jews. Data on laterality were not available for large series in any other part of the world. Bilateral tumours occurred with almost exactly equal frequency in boys and girls (M/F = 1.02). They tended to occur slightly earlier than unilateral Wilms' tumour. Only two series, United States SEER Whites and England and Wales, had 10 or more bilateral cases. In the American series, 50% of bilateral cases were aged under 2 years compared with 34% of unilateral, while in England and Wales 58% of bilateral and 32% of unilateral cases occurred before age 2. No cases of bilateral Wilms' tumour above age 8 were recorded from any registry in the study.

*Renal carcinoma*

Renal carcinoma was everywhere rare in children, and no large series had an ASR greater than 0.2 per million. In Europe, carcinomas accounted for 1.5–3% of all childhood renal tumours. Similar proportions were observed in United States Whites and Blacks. Among Chinese populations, where Wilms' tumour had a lower incidence, carcinoma accounted for 10% of all childhood kidney tumours but its incidence was similar to that in other regions. Figure 3 shows the distribution by age and sex of all 153 registrations for renal carcinoma in the study. Only eight cases (5%) were not stated to be histologically verified. The numbers of cases at each year of age were similar until around age 12, with a moderate rise thereafter. Renal carcinoma was registered approximately twice as frequently in boys as in girls during the first decade of life, but equal numbers of cases occurred in the two sexes among children aged 10 and over.

### Discussion

Since the publication of Innis's paper in 1972, the view that Wilms' tumour is an 'index cancer of childhood' with approximately constant incidence worldwide has gained wide currency (Davies, 1976; Breslow & Beckwith, 1982; Lucas & Fischer, 1990). It is clear, however, from the results of the present study that there is a considerable variation in the incidence of Wilms' tumour between different regions and ethnic groups.

The highest rates in this study were found among Blacks, in both Africa and the United States. Blacks were on average 3.6 months older than Whites in the United States National Wilms' Tumour Study (NWTS), which included 528 Black children (Breslow *et al.*, 1988). We could find little evidence for any difference but there were only 163 United States Blacks in the present study. The incidence in predominantly White Caucasian populations was lower than for Blacks and was similar in all regions from which data were available. The lowest incidence was found in Asia, and especially in the eastern part of that continent, though the area of low incidence appears to extend at least as far west as India. In many of the east Asian series, Wilms' tumour occurred at a much earlier age than elsewhere, indeed the highest incidence was found in infancy. Incidence rates for Wilms' tumour were not available for children of East Asian ethnic groups in the United States, but the Asian children in the NWTS had a mean age of 29.1 months compared with 43.7 months for Whites (Breslow *et al.*, 1988). The ASR for all childhood renal tumours among Asians in Los Angeles, San Francisco and Hawaii during 1972–82 was 2.2 per million (Waterhouse *et al.*, 1982; Muir *et al.*, 1987) though this was based on only four cases. During 1960–84, the incidence of renal tumours in Asian children in Hawaii was less than two thirds of that in Whites (Goodman *et al.*, 1989).

In Israel the relationship between the rates for Jews and non-Jews is hard to interpret. In the present study, the non-Jews appeared to follow the Asian pattern, while the incidence among Jews was similar to that in White Caucasians. The rate for non-Jews was, however, based on small numbers, and in a previous series covering 1961–65, with a total of 35 cases of Wilms' tumour, Israeli Arabs had an ASR of 16 per million, twice that of Jews (Virag & Modan, 1969). Underlying risks to the predominantly Arab, non-Jewish population may have changed with time, but combining the results for the two periods produces a rate very similar to that for Jews.

The results presented here, though based on small numbers of cases, suggest that Wilms' tumour is relatively common among the indigenous peoples of Oceania. The Hawaiian ethnic group in Hawaii, however, had a low incidence of childhood renal tumours but this was again based on only eight cases (Goodman *et al.*, 1989).

Some cases of Wilms' tumour have been explicitly described as heritable in origin. These include bilateral tumours, those which occur in association with aniridia and certain other congenital abnormalities, and the small number of cases which form part of familial aggregations. Fewer than one in 15 cases of Wilms' tumour are bilateral and the other classes of 'genetic' Wilms' tumour account for even smaller proportions (Breslow & Beckwith, 1982; Pastore *et al.*, 1988). The frequency of associated congenital abnormalities is higher in Blacks, who have a higher incidence of Wilms' tumour, than in Whites (Kramer *et al.*, 1984). The genetic damage which is postulated to give rise to Wilms' tumour in some cases could of course itself be caused by environmental factors. Aetiological factors for Wilms' tumour have been investigated in many studies, sometimes as part of larger studies of all childhood cancers. Much attention has been focused on various occupational associations but these have generally only been found in a small proportion of all studies (Arundel & Kinnier Wilson, 1986; Bunin *et al.*, 1989). The age distribution of Wilms' tumour is strongly suggestive of pre-natal origins. The lack of consistency in reports of environmental risk factors together with the ethnic variations and genetic associations described above strongly suggest that the risk of Wilms' tumour may be predominantly genetically determined at the population level with predisposition varying between populations of different ethnic origin, though the familial element appears to be small.

The series of Wilms' tumour reported here will have included small numbers of cases of two other tumours which are now regarded as distinct entities. The first of these is the bone-metastasising renal tumour of childhood or clear cell sarcoma of the kidney. This is a rare tumour, accounting for around 4–5% of cases in clinical trials (Marsden *et al.*, 1984; D'Angio *et al.*, 1989). It has a similar age distribution to Wilms' tumour but occurs very much more frequently in boys than in girls; in the largest reported series the sex ratio was M/F = 6.6:1 (Marsden & Lawler, 1980). We could find no published references to the aetiology of this tumour. The second rare tumour now distinguished from Wilms' tumour is the rhabdoid renal tumour, which accounts for around 2% of all tumours formerly classified as Wilms' (D'Angio *et al.*, 1989). Rhabdoid renal tumour tends to occur in younger children than Wilms' tumour, with a median age of 1 year in the NWTS series of over 100 cases (Weeks *et al.*, 1989). The sex ratio in the NWTS series was M/F = 1.47:1. The association of rhabdoid tumour with medulloblastoma and other embryologically unrelated brain tumours in the same patient is well documented (Bonnin *et al.*, 1984; Weeks *et al.*, 1989), suggesting that there may be a large heritable component to its aetiology. Nothing is known of international variations in the incidence of either of these tumours.

Another type of renal tumour seen predominantly in very young children is the mesoblastic nephroma. This is not a malignant tumour, and thus it is not generally recorded systematically by cancer registries. The Manchester Children's Tumour Registry ascertained five cases over a 30-year period (Marsden & Newton, 1986). All were in infants aged under 6 months, and all but one aged under 3 months. They accounted for half of all renal tumours in children aged under 6 months, and 17% of those under 1 year of age. Many tumours at one time described as Wilms' in infants would in fact have been mesoblastic nephromas (Bolande, 1974). It was not possible to tell how far this phenomenon contributed to the apparent excess of Wilms' tumour in infants in east Asian registries, but of the 32 infants registered in Osaka with Wilms' tumour only four (13%) were aged under 3 months, suggesting that few of these tumours were really mesoblastic nephromas. The markedly lower average age for American Asians (of mostly east Asian extraction) in the NWTS, in which the pathology was reviewed centrally, also suggests that the excess of Wilms' tumour among Asian infants is real.

Carcinoma of the kidney is predominantly a disease of adults. There was little sign of any variation in incidence rates in childhood in the present study, with very low rates in all regions. There were roughly constant numbers of cases at each year of age below 12, after which point there could be observed the start of the steady increase in incidence which continues through early adulthood. In the first decade of life there were twice as many boys affected as girls, whereas among older children there was a slight excess of girls. These patterns contrast with those for Wilms' tumour, which occurs largely in the first 5 years of life and is equally common in the two sexes.

Patterns of incidence for renal cancer (mainly carcinoma) in adults can be found in *Cancer Incidence in Five Continents* (Muir *et al.*, 1987). From the truncated standardised rates (for persons aged 35–64), it seems that the disease is in general about twice as common in males as females. The lowest rates are recorded in Asia, particularly in India but also in Japanese and Chinese populations. Rates in North America and the Nordic countries are somewhat higher than those for other parts of Europe. In the United States, the incidence is similar for Blacks and Whites. These patterns contrast with the recorded incidence of renal carcinoma in later childhood as regards both sex ratio and geographical distribution. This suggests either that renal carcinoma in children has a different aetiology from tumours of the same morphology in adults, or that some of the childhood cases were misclassified. It is not clear, however, why Wilms' tumours should be more likely to be miscoded as carcinomas if they occur in boys rather than girls.

While incidence of Wilms' tumour varies predominantly by ethnic group rather than geographically, suggesting that genetic predisposition is important in its aetiology, the causes of renal carcinoma in childhood remain more completely a mystery.

Many of the data on which this paper is based are presented in *International Incidence of Childhood Cancer* (Parkin *et al.*, 1988*a*) and we wish to acknowledge the contributions to that volume of our co-editors, Dr G.J. Draper, Mr C.A. Bieber, Dr B. Terracini and Dr J.L. Young. Our particular thanks go to all the contributors to that monograph, for whose contributions it was not practicable to give individual references. We are grateful to Mrs E.M. Roberts for secretarial help and to Mr J. Ferlay and Mr E. Masuyer for work on the figures and other computing. The Childhood Cancer Research Group is supported by the Department of Health and the Scottish Home and Health Department.

### References

ARUNDEL, S.E. & KINNIER WILSON, L.M. (1986). Parental occupations and cancer: a review of the literature. *J. Epidemiol. Comm. Health*, **40**, 30.

BIRCH, J.M. & MARSDEN, H.B. (1987). A classification scheme for childhood cancer. *Int. J. Cancer*, **40**, 620.

BOLANDE, R.P. (1974). Congenital and infantile neoplasia of the kidney. *Lancet*, **ii**, 1497.

BONNIN, J.M., RUBINSTEIN, L.J., PALMER, N.F. & BECKWITH, J.B. (1984). The association of embryonal tumours originating in the kidney and in the brain. *Cancer*, **54**, 2137.

BRESLOW, N.E. & BECKWITH, J.B. (1982). Epidemiological features of Wilms' tumor: results of the National Wilms' Tumor Study. *J. Natl Cancer Inst.*, **68**, 429.

BRESLOW, N.E., BECKWITH, J.B., CIOL, M. & SHARPLES, K. (1988). Age distribution of Wilms' tumour: report from the National Wilms' Tumour Study. *Cancer Res.*, **48**, 1653.

BUNIN, G.R., NASS, C.C., KRAMER, S. & MEADOWS, A.T. (1989). Parental occupation and Wilms' tumour: results of a case–control study. *Cancer Res.*, **49**, 725.

DAVIES, J.N.P. (1968). Some variations in childhood cancers throughout the world. In *Tumours in Children*, Marsden, H.B. & Steward, J.K. (eds) p. 13. Springer-Verlag: Berlin.

DOLL, R. & SMITH, P.G. (1982). Comparison between registries; age-standardised rates. In *Cancer Incidence in Five Continents. Vol. IV.* Waterhouse, J., Muir, C.S., Shanmugaratnam, K. & Powell, J. (eds) p. 671. IARC: Lyon.

D'ANGIO, G.J., BRESLOW, N., BECKWITH, J.B. & 10 others (1989). Treatment of Wilms' tumor. Results of the Third National Wilms' Tumor Study. *Cancer*, **64**, 349.

GOODMAN, M.T., YOSHIZAWA, C.N. & KOLONEL, L.N. (1989). Ethnic patterns of childhood cancer in Hawaii between 1960 and 1984. *Cancer*, **64**, 1758.

INNIS, M.D. (1972). Nephroblastoma: possible index cancer of childhood. *Med. J. Aust.*, **1**, 18.

KRAMER, S., MEADOWS, A.T. & JARRETT, P. (1984). Racial variation in incidence of Wilms' tumor: relationship to congenital anomalies. *Med. Pediatr. Oncol.*, **12**, 401.

LUCAS, S.B. & FISCHER, P.R. (1990). No neuroblastoma in Zaire (letter). *Lancet*, **i**, 115.

MARSDEN, H.B. & LAWLER, W. (1980). Bone metastasizing renal tumour in childhood: histological and clinical review of 38 cases. *Virchows Arch. A. Path. Anat.*, **387**, 341.

MARSDEN, H.B., LAWLER, W., CARR, T. & KUMAR, S. (1984). A scoring system for Wilms' tumour: pathological study of the second Medical Research Council (MRC) trial. *Int. J. Cancer*, **33**, 365.

MARSDEN, H.B. & NEWTON, W.A. (1986). New look at mesoblastic nephroma. *J. Clin. Pathol.*, **39**, 508.

MUIR, C., WATERHOUSE, J., MACK, T., POWELL, J. & WHELAN, S. (eds) (1987). *Cancer Incidence in Five Continents. Vol. V.* IARC: Lyon.

PARKIN, D.M., STILLER, C.A., BIEBER, A., DRAPER, G.J., TERRACINI, B. & YOUNG, J.L. (eds) (1988*a*). *International Incidence of Childhood Cancer.* IARC: Lyon.

PARKIN, D.M., STILLER, C.A., DRAPER, G.J. & BIEBER, C.A. (1988*b*). The international incidence of childhood cancer. *Int. J. Cancer*, **42**, 511.

PASTORE, G., CARLI, M., LEMERLE, J. & 8 others (1988). Epidemiological features of Wilms' tumor: results of studies by the International Society of Paediatric Oncology (SIOP). *Med. Pediatr. Oncol.*, **16**, 7.

VIRAG, I. & MODAN, B. (1969). Epidemiologic aspects of neoplastic diseases in Israeli immigrant population: II, Malignant neoplasms in childhood. *Cancer*, **23**, 137.

WATERHOUSE, J., MUIR, C., SHANMUGARATNAM, K. & POWELL, J. (eds) (1982). *Cancer Incidence in Five Continents. Vol. IV.* IARC: Lyon.

WEEKS, D.A., BECKWITH, J.B., MIERAU, G.W. & LUCKEY, D.W. (1989). Rhabdoid tumor of kidney: a report of 111 cases from the National Wilms' Tumor Study pathology center. *Am. J. Surg. Pathol.*, **13**, 439.